# Therapeutic Potential of Exosomes Derived From circRNA_0002113 Lacking Mesenchymal Stem Cells in Myocardial Infarction

**DOI:** 10.3389/fcell.2021.779524

**Published:** 2022-01-19

**Authors:** Tiantian Tian, Feng Li, Ruihua Chen, Zhiwei Wang, Xueming Su, Chao Yang

**Affiliations:** ^1^ Hainan Yiling Medical Industry Development Company Ltd., Qionghai, China; ^2^ Center for Biological Science and Technology, Beijing Normal University, Zhuhai, China; ^3^ State Key Laboratory of Quality Research in Chinese Medicine, Macau University of Science and Technology, Taipa, China

**Keywords:** circRNA_0002113, exosomes, MSCs, myocardial infarction, Runx1, miR-188-3p

## Abstract

Exosomes are participated in the pathogenesis of cardiovascular diseases and can be secreted by mesenchymal stem cells (MSCs). However, the effects of circRNA, delivered by exosomes derived from MSCs, on myocardial injury remain unclear. Hence, this study aims to explore the therapeutic potential of exosomes derived from circRNA_0002113 lacking MSCs in the treatment of myocardial injury *in vitro* and *in vivo*. Our results reveal that exosomes derived from circRNA_0002113 lacking MSCs decreased cell apoptosis in anoxia-reoxygenation (A/R) model cells, and reduced myocardial injury by inhibiting nuclear translocation of RUNX1 *in vitro* and *in vivo*. Moreover, miR-188-3p, which targets RUNX1 in cardiomyocytes was also found to interact with circRNA_0002113. In conclusion, exosomes derived from circRNA_0002113 lacking MSCs could suppress myocardial infarction by sponging miR-188-3p to regulate RUNX1 nuclear translocation. The circRNA_0002113/miR-188-3p/RUNX1 axis mediated alleviation of apoptosis serves as a novel strategy to treat myocardial I/R injury.

## Introduction

Myocardial infarction generally refers to myocardial necrosis caused by acute and persistent coronary ischemia (Osman et al., 2020). Myocardial infarction mainly occurs via two pathways: 1.) coronary spasm that dramatically increases the myocardial oxygen consumption, 2.) coronary atherosclerosis obstructing coronary lumen, which leads to myocardial ischemic necrosis (Ge et al., 2019). At present, the primary clinical methods for treating myocardial infarction include drug therapy, coronary intervention, and coronary artery bypass grafting (Matteucci et al., 2019; Kupó et al., 2020). Although these measures can alleviate the initial myocardial damage in the acute phase, heart failure-derived cardiac remodeling negatively affects the treatment of patients with myocardial infarction (Tzahor and Poss, 2017; Bertero and Maack, 2018; Shah et al., 2019). Therefore, novel regenerative strategies for the infarcted myocardium remain to be explored.

Mesenchymal stem cells (MSCs) are a class of cells with multiple phenotypes that can self-renew and proliferate *in vivo*. MSCs have a wide variety of applications namely, reducing scars and left ventricular remodeling, promoting angiogenesis, regulating inflammatory response, repairing myocardial tissue, and improving cardiac function after myocardial infarction (Khan et al., 2017; Luger et al., 2017; Abushouk et al., 2019). However, the MSCs transplantation efficiency is limited by the low rate of cell retention in the treatment of myocardial infarction (Chen et al., 2018). Current studies suggest that MSCs exert their protective effects on myocardial infarction *via* a paracrine mechanism, rather than directly differentiating into cardiomyocytes or vascular endothelial cells, which promote proliferation and survival of cardiomyocytes and angiogenesis and thereby improve cardiac function (Tang et al., 2016). Exosomes are the biologically active components of MSCs and play a major role in the paracrine effects of MSCs (Marote et al., 2016; Phinney and Pittenger, 2017; Zhang et al., 2019). Different cell types secrete exosomes, which contain protein receptors, lipid mRNA, non-coding RNAs, and transcription factors, under normal or pathological conditions (Boilard, 2018; Ghafarian et al., 2019). Exosomes released from specific cells are transported to different cells and affect the physiological pathways in recipient cells (Babaei and Rezaie, 2021). MSCs derived exosomes contain cytoprotective factors such as growth factors, which can resist apoptosis and fibrosis, enhance myocardial differentiation and repair damaged tissue (Yoshida et al., 2018; Deng et al., 2019; Nikfarjam et al., 2020; Salimi et al., 2020). Giricz et al. found that ischemic preconditioning can induce secretion of exosomes, thereby making the myocardium resistant to a subsequent ischemic insult and hence reducing the area of myocardial infarction (Giricz et al., 2014).

Circular RNAs (circRNAs) are a special type of non-coding RNAs that have a covalently closed loop structure and regulate gene expression by interacting with microRNA (miRNA) (Su and Lv, 2020). CircRNAs have been confirmed to play a crucial role in cardiovascular diseases (Kristensen et al., 2019; Lu and Thum, 2019). A recent study has shown that ciRS-7, a circRNA, aggravates myocardial cell apoptosis after myocardial infarction and up-regulates the expression of PARP and SP1 by inhibiting miR-7 (Holdt et al., 2016). However, the relationship between circRNA and exosomes isolated from MSCs and their function in myocardial infarction remains unclear. In this study, we found that circRNA_0002113 has up-regulated in anoxia-reoxygenation (A/R) cells. Exosomes isolated from circRNA_0002113 lacking MSCs reduced the apoptosis in A/R model cells. Moreover, circRNA_0002113 modulated RUNX1 nuclear translocation by sponging miR-188-3p in A/R cells. These data revealed the therapeutic effects of exosomes derived from circRNA_0002113 lacking MSCs in myocardial infarction by sponging miR-188-3p to regulate RUNX1 nuclear translocation.

## Materials and Methods

### Culture of Rat Cardiac Cells

H9C2 cells were purchased from the Shanghai Institute of Chinese Academy of Sciences (Shanghai, China). The cells were cultured in Dulbecco’s Modified Eagle Medium (DMEM; Gibco) with 10% fetal bovine serum (FBS; Gibco) at 37°C in a humidified atmosphere with 5% CO_2_.

### Isolation and Culture of Bone Marrow-Derived MSCs

The rats were anesthetized and sacrificed. Tibias and femurs were acquired under sterile conditions, and the bone marrow was flushed with Phosphate-buffered saline (PBS) supplemented with 2% FBS. The cell suspension was filtered through a 40-μm cell strainer (BD Falcon, Franklin Lakes, NJ, United States). After centrifugation of the filtered suspension, the bone marrow cells were cultured in DMEM/F12 (Dulbecco’s Modified Eagle Medium/Nutrient Mixture F-12) (HyClone, Beijing, China) supplemented with 10% FBS.

### Isolation and Identification of MSCs-Derived Exosomes

Exosomes were isolated from the bone marrow-derived MSCs supernatant. Briefly, the supernatant was centrifuged to remove cellular debris. The supernatant obtained was mixed with the Total Exosome Isolation Reagent (Invitrogen, United States) and kept overnight at 4°C. After centrifuging at 10,000 g at 4°C for 1 h, the pellet was re-suspended in 200 μL of PBS and stored at −80°C. The exosome morphology from pLVXcirc treated MSCs was observed with a transmission electron microscope (TEM, JEOL). Dynamic light scattering (DLS) measurements were performed by the Malvern Zetasizer Nano ZS (Malvern, Herrenberg, Germany). The surface charges of pLVXcirc treated MSC-derived exosomes were determined from 1 to 4 days’ preservation at 4°C using the Zeta Potential Analyzer (Brookhaven Instrument Co., CA).

### Anoxia-Reoxygenation Model

Two hours before the test, the medium of H9C2 cells was replaced with a glucose-free medium without growth factors or serum. Cell plates with 5×10^5^ density were placed in a glass chamber with 95% N_2_/5% CO_2_ gas for 1 h, which mimics anoxic condition, followed by reoxygenation (95% O_2_, 5% CO_2_) for different time points: 1, 2, 4, 6, and 8 h respectively.

### Cell Viability Assay

1 × 10^4^ H^9^C2 cells were cultured in 96-well plates. At different time points of the A/R model, 10 μL of the Cell Counting Kit-8 (CCK-8) solution (Dojindo, Japan) was added to each well. The cells were incubated for another 4 h at 37°C. The Optical Density (OD) values at 450 nm were measured using a microplate reader (ELx800, BioTeK, United States).

### Quantitative RT-PCR

Total RNA was extracted from H9C2 cells using the TRIzol reagent (Invitrogen, Carlsbad, CA, United States). The RNA was reverse transcribed to cDNA using PrimeScript RT Master Mix Kit or PrimeScript miRNA cDNA Synthesis Kit (Takara Bio). SYBR Green PCR Master Mix (Invitrogen) was used to assess the expression, using a 7900HT real-time PCR system (Applied Biosystems, Life Technologies). U6 snRNA and GAPDH were used as internal controls. Fold changes in the expression levels were calculated using the comparative cycle threshold (CT) method (2^−ΔΔCt^). The real-time quantitative RT-PCR was performed in triplicates. The primers were as follows: circ_0002113 forward: 5′-TAA​ATG​GTT​CAC​GGC​CGA​CAT-3′; circ_0002113 reverse: 5′- ATC​TGG​CAT​GGC​TGA​CTA​CG-3′; miR-188-3p forward: 5′- ATT​ATT​GGC​TCC​CAC​ATG​CAG​GG-3′; miR-188-3p reverse: 5′- ATC​CAG​TGC​AGG​GTC​CGA​GG-3′; RUNX1 forward: 5′-CCT​CCT​TGA​ACC​ACT​CCA​CT-3′; RUNX1 reverse: 5′- CTG​GAT​CTG​CCT​GGC​ATC-3′; GAPDH forward: 5′-ATG​ACT​CTA​CCC​ACG​GCA​AG-3′; reverse: 5′- CTG​GAA​GAT​GGT​GAT​GGG​TT-3′; U6 forward: 5′-TGC​TTC​GGC​AGC​ACA​TAT​AC-3′; reverse: 5′- AGG​GGC​CAT​GCT​AAT​CTT​CT-3′.

### Exosomes Treatment and Cell Fraction Protein Extraction

To detect the effects of exosomes on A/R H9C2 cells *in vitro*, A/R cells were treated with pLVXcirc transfected MSC-derived exosomes (10 μg/ml) for 24 h H9C2 cells were resuspended in lysis buffer (10 mM HEPES, 10 mM NaCl, 1 mM KH_2_PO_4_, 5 mM NaHCO_3_, 5 mM EDTA, 1 mM CaCl_2_, 0.5 mM MgCl_2_ and protease inhibitors), and the samples were centrifuged at 6300 g for 5 min at 4°C. The supernatant was collected as the cytosolic fraction. The pellet was washed, and the nuclei were lysed using TSE buffer (10 mM Tris-HCl, 300 mM sucrose, 1 mM EDTA, 0.1% IGEPAL-CA 630 (v/v), pH 7.5). 100 mg heart tissues were rinsed with ice-cold PBS containing lysis buffer (50 mM Tris, pH 7.4, 150 mM NaCl, 1 mM EDTA, 1% Nonidet P-40, 0.1% SDS and protease inhibitors). The tissue was then dissected with a tissue grinder 30 times. The samples were centrifuged at 12,000 g for 10 min at 4°C. The supernatant was collected as the cytosolic fraction. Nuclear proteins were extracted using a TSE buffer.

### Western Blotting

The protein concentration was quantified and then proteins were transferred onto nitrocellulose membranes. The nitrocellulose membranes were blocked and incubated with the primary antibody for 2 h at room temperature. The primary antibodies against RUNX1 (Abcam), USP7 (Cell Signaling Technology), USP10 (Cell Signaling Technology), p53 (Cell Signaling Technology), Bax (Cell Signaling Technology), CD9 (Abcam), CD63 (Abcam), and CD81 (Abcam) were utilized. Next, the nitrocellulose membranes were incubated with the secondary antibody (anti-rabbit IgG, Cell Signaling Technology) and GAPDH antibody (Cell Signaling Technology). Finally, the protein expression was visualized using the enhanced chemiluminescence (ECL) detection system.

### Northern Blotting

Total RNA was isolated from H9C2 cells using the TRIzol reagent (Invitrogen, Carlsbad, CA, United States). Circ_0002113 probe for northern blotting was acquired using the Biotin RNA labeling mix (Roche). The RNA samples were separated by electrophoresis and were then transferred onto nitrocellulose membranes. The nitrocellulose membranes were incubated with the hydration buffer containing the probes. Finally, the RNA signal was visualized using the chemiluminescence detection system.

### miRNA, circRNA, siRNA, Lentivirus Construction and Transfection

The miR-188-3p mimic, control mimic, circ_0002113 specific shRNA, si-RUNX1, and si-USP7 were obtained from Sangon Biotech (Shanghai, China). Circ_0002113 specific shRNA was cloned into a pLVX-shRNA vector (Clontech, #632179). CircRNA overexpression vector (#GS0108) was obtained from Geneseed (Guangzhou, China). 50 nM miR-188-3p mimic or control mimic, and 50 nM si-RUNX1, si-USP7 or negative control siRNA (si-NC) were used for cell transfection by Lipofectamine RNAiMAX (Invitrogen) or Lipofectamine 3000 Reagent (Invitrogen) in H9C2 cells as per the manufacturer’s instruction. Five groups were indicated *in vitro* experiments: H9C2 cells without treatments as a control group; A/R H9C2 cells group; A/R + MSCs-exo group: A/R cells were treated with MSC-derived exosomes; A/R + pLVXcirc group: A/R cells were treated with pLVXcirc; A/R + MSCs-pLVXcirc-exo group: A/R cells were treated with pLVXcirc transfected MSC-derived exosomes.

### Bioinformatics Analysis

The potential interaction of circ_0002113 with miR-188-3p was predicted by the CircInteractome program. The putative targets of miR-188-3p were determined using the TargetScan.

### RNA Pull-Down Analysis

Biotin-labeled miR-188-3p and biotin labeled negative control (NC) (GenePharma, Shanghai) were incubated with magnetic beads (Life Technologies, Carlsbad, CA, United States) at 37°C for 4 h. For miR-188-3p/circ_0002113 and miR-188-3p/RUNX1 pull-down analysis, H9C2 cells were transfected with biotin-miR-188-3p or biotin-NC using Lipofectamine 3000 Reagent (Invitrogen). The cells were lysed and incubated with magnetic beads (Life Technologies, Carlsbad, CA, United States) at 4°C overnight. After washing with wash buffer, circ_0002113 and RUNX1 in the pull-down were determined using RT-PCR analysis.

### Dual-Luciferase Reporter Assay

To determine the relationship between RUNX1 and miR-188-3p, wild-type (*wt*) and mutant (*mut*) vectors containing the 3′-UTR of RUNX1 were cloned into pGL3 luciferase reporter plasmids (Promega, Madison, United States). 100 ng of plasmids with *wt* or *mut* 3′-UTR RUNX1 sequences and 10 ng of pRL-TK Renilla luciferase reporter were co-transfected with 10 nM miR-188-3p mimic or negative control mimic (Sangon Biotech) into H9C2 cells. For circ_0002113 and miR-188-3p luciferase reporter assay, the circ_0002113 sequences containing *wt* or *mut* binding sites were respectively inserted into psiCHECK2 luciferase vector (Promega), and then co-transfected with 10 nM miR-188-3p mimic or negative control mimic into H9C2 cells. After cultivation at 37°C for 24 h, cells were assessed using the dual-luciferase reporter assay system (Promega, Madison, United States) as per the manufacturer’s instructions. Relative luciferase activity was normalized to the Renilla luciferase internal control.

### TUNEL Assay

H9C2 cell apoptosis was measured using the TUNEL assay (terminal deoxynucleotidyl transferase dUTP nick end labeling) (Roche, Basel, Switzerland) as per the manufacturer’s protocols. The cells with TUNEL-labeled nuclei were regarded as TUNEL-positive cells. The representative staining (200x) was used to reveal the TUNEL-positive cells. The quantification of TUNEL-positive cells was performed using the ImageJ software.

### Chip Assay

H9C2 cells were fixed using 1% formaldehyde in PBS and chromatin was cross-linked for 15 min at 37°C. Crosslinking reaction was terminated by the addition of 125 mM glycine for 5 min at room temperature. Cells were washed with PBS, lysed, and the chromatin was solubilized to the approximately 150–600 base pairs (bp) length by sonication. Immunoprecipitation of the cross-linked proteins was observed by the addition of anti-USP7 (Cell Signaling Technology; 2 μg). The IgG antibody (1 μg) served as the control. Immunoprecipitates were analyzed by detecting p53 expression.

### Co-Immunoprecipitation Assay

For Co-IP experiments, anti-USP7 was added to the H9C2 cell protein extracts. The mixture was then incubated with protein A/G magnetic beads overnight at 4°C. The immunoprecipitates were resolved by SDS-PAGE and analyzed by immunoblotting with indicated antibodies.

### Animals

6–8 weeks male SD rats weighing 180–220 g were acquired from Second Military Medical University (Shanghai, China) and maintained under specific pathogen-free conditions. All animals were treated according to the guidelines for the Care and Use of Laboratory Animals. Guidelines of the National Academy of Sciences and published by the National Institutes of Health were followed for all the animal experiments. All animal experimental protocols were approved by the Institutional Animal Care and Use Committee of the Second Military Medical University (No.20200065).

### I/R Animal Model and Treatment

Rats were subjected to ischemic conditions for 45 min, followed by reperfusion for 3 h. The rats were anesthetized, the chest was opened to expose the heart and identify the left anterior descending coronary artery (LAD). We passed a silk suture around the LAD at the inferior border of the left auricle and occluded the artery by snaring with a vinyl tube through which the ligature had been passed. We occluded the coronary artery by pulling the snare tight and securing it with a hemostat. After 45 min of ischemia, we released the ligature and reperfused the heart. The Sham-operated group experienced the same procedure except that the snare was left untied. The exosome treatment group immediately received an exosome transplantation (20 mg/kg) or pLVXcirc (20 mg/kg) through the tail vein in 200 μL PBS. Rats (*n* = 25) were randomly assigned to five groups (*n* = 5 per group): 1) sham group as control; 2) I/R group: I/R model rats without exosome or pLVXcirc treatment. 3) I/R + MSCs-exo group: I/R rats were treated with MSC-derived exosomes for five consecutive days; 4) I/R + pLVXcirc group: I/R rats were treated with pLVXcirc for five consecutive days; 5) I/R + MSCs-pLVXcirc-exo group: I/R rats were treated with pLVXcirc transfected MSC-derived exosomes for five consecutive days. After 28 days, the animals were sacrificed to evaluate the myocardial injury.

### Myocardial Infarct Size Measurements

Myocardial infarct size was detected by triphenyl tetrazolium chloride (TTC) staining and Evans blue dye staining. The hearts were cut into 2 mm thick slices. The slices were stained with 1% TTC at room temperature for 30 min. The area stained blue by Evans blue represents the area, not at risk. The infarcted myocardium was not stained with any dye and hence appeared whiter than other areas.

### Measurements of Cardiac Troponin-T

The cTn-T in serum was quantified using the cTn-T ELISA kit (Abbkine) as per the manufacturer’s protocols. The OD values were assessed using a microplate reader (ELx800, BioTeK, United States). The concentration of cTn-T was determined using the standard curve.

### Immunofluorescence Analysis

H9C2 cells were fixed in 4% paraformaldehyde (PFA) for 10 min and permeabilized using 0.2% Triton X-100. The cells were blocked using 10% bovine serum albumin (BSA) for 1 h and then incubated with anti-RUNX1 (1:200, Abcam) primary antibody at 4°C overnight. Further, the cells were washed with PBS 0.05% Tween (PBST) followed by incubation with Alexa Fluor594 antibody (1:1000, Abcam). After 1 h of incubation at 37°C, cell nuclei were stained with DAPI. Images were obtained by fluorescence microscope (Leica AF6000). The quantification of RUNX1 positive cells was performed using the ImageJ software.

The heart was dissected and then fixed in 4% PFA for 4 h. The heart sections (5 µm) were embedded in polyethylene glycol. USP7 (1:100; Cell Signaling Technology) and p53 (1:500; Cell Signaling Technology) antibodies were incubated to evaluate the interaction of USP7 and p53 in I/R rats. The sections were then incubated with Alexa Fluor 488 antibody (1:1000; Abcam) or Alexa Fluor594 antibody (1:1000, Abcam) for 2 h at 37°C. The nuclei were stained with DAPI. The images were visualized using Aconfocal microscopy (Zeiss 710).

### Statistical Analysis

Data from three independent experiments were represented as Mean ± standard deviation (SD). Differences between the two groups were analyzed using the Student’s t-test or ANOVA followed by Dunnett’s test. A *p*-value of <0.05 was considered to be statistically significant.

## Results

### CircRNA_0002113, RUNX1, and miR-188-3p Expression in A/R Model Cells

**FIGURE 1:**
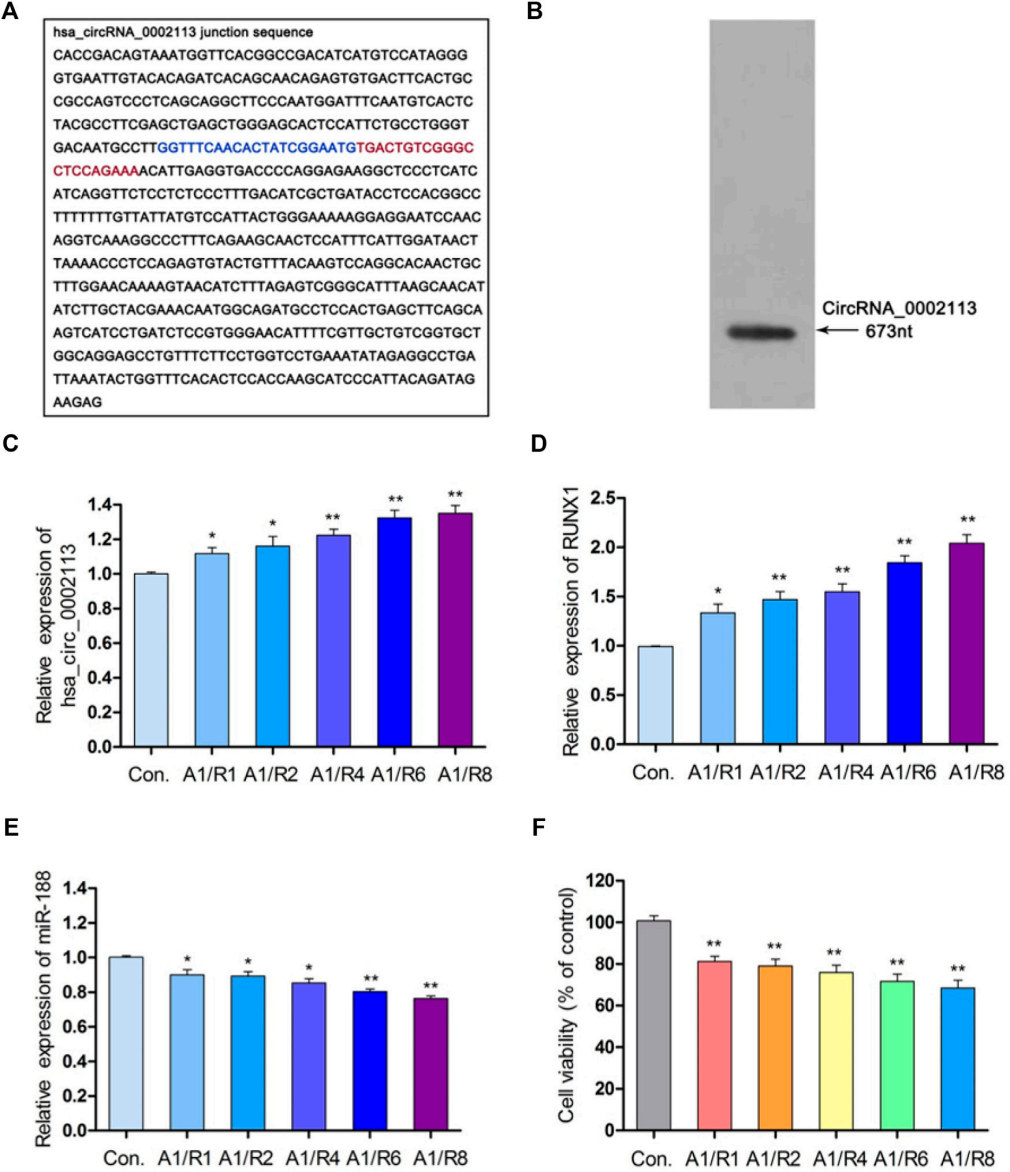
CircRNA_0002113 expression was induced in the A/R model. **(A)** The spliced sequences of circRNA_0002113. The junction site was located between the blue and red base. **(B)** CircRNA_0002113 sequence was validated by northern blotting. **(C)** The expression of circRNA_0002113 was evaluated after 1 h of anoxia followed by reoxygenation for 1, 2, 4, 6, 8 h. **(D)** The expression of miR-188-3p was evaluated after 1 h of anoxia followed by reoxygenation for 1, 2, 4, 6, 8 h. **(E)** The expression of RUNX1 was evaluated after 1 h of anoxia followed by reoxygenation for1, 2, 4, 6, 8 h **(F)** H9C2 cell viability was determined after 1 h of anoxia followed by 1, 2, 4, 6, 8 h of reoxygenation. *p < 0.05, **p < 0.01, compared with the control. A, anoxia; R, reoxygenation.

Interferon-gamma receptor 2 (IFNGR2), the parent gene of circRNA_0002113, was reported to be involved in the progress of myocardial infarction (Tiroch et al., 2005; Shalia et al., 2017). Figures 1A,B showed the spliced circRNA_0002113 sequence and the Northern blot verification results of the splicing site of circRNA_0002113, respectively. To investigate the effect of circRNA_0002113 on myocardial infarction *in vitro*, the A/R model was constructed using H9C2 cells and the expression levels of circRNA_0002113 were evaluated after 1, 2, 4, 6, 8 h of reoxygenation preceded by 1 h of anoxia. The results indicate that circRNA_0002113 expression significantly increases after reoxygenation preceded by anoxia when compared with the control (Figure 1C). Interestingly, the expression levels of RUNX1 also increase in the A/R model, similar to the circRNA_0002113 patterns (Figure 1D). In contrast, miR-188-3p expression decreases dramatically after reoxygenation preceded by anoxia when compared with the control (Figure 1E). To identify the ideal reoxygenation time, cell viability at different time durations was assessed. The results reveal that the viability of H9C2 cells was significantly reduced after reoxygenation preceded by anoxia when compared with the control (Figure 1F).

### Exosomes Derived From pLVX-circRNA_0002113-shRNA Treated MSCs Reduced Apoptosis of A/R H9C2 Cells

**FIGURE 2:**
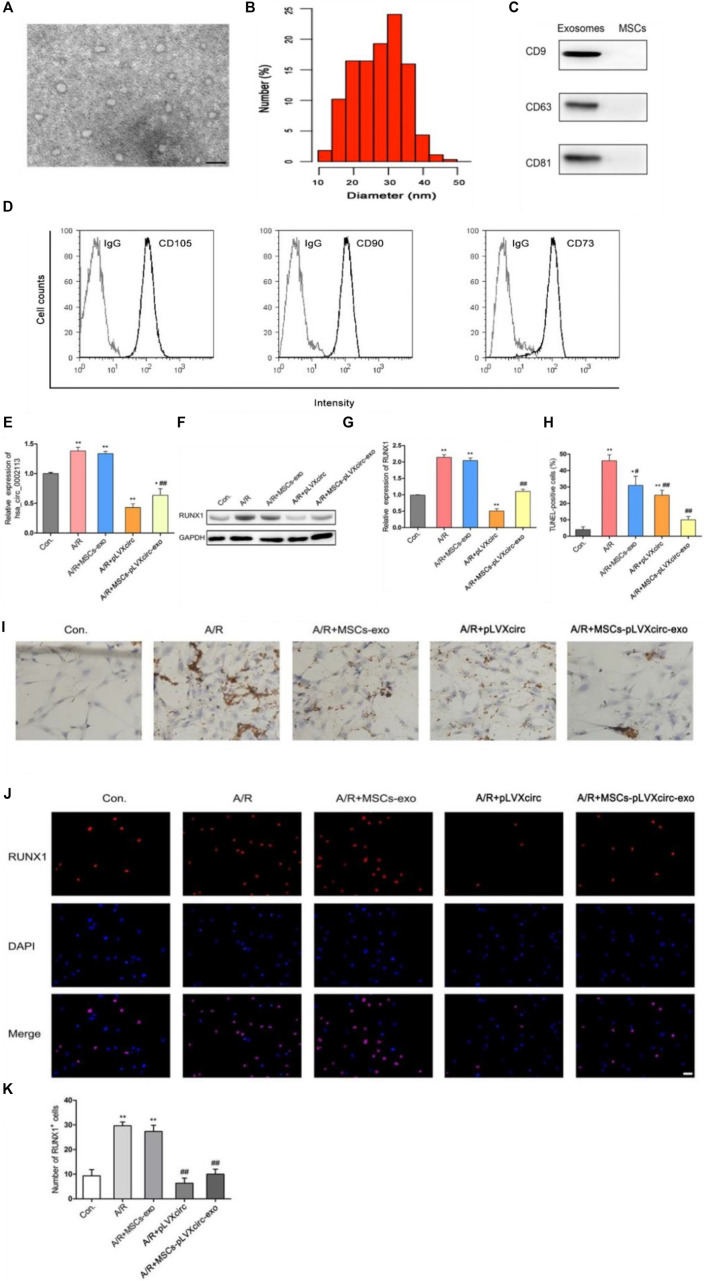
Exosomes derived from pLVXcirc treated MSCs decreased apoptosis in A/R H9C2 cells. **(A)** Transmission electron microscope (TEM) of exosomes isolated from pLVXcirc treated MSCs. **(B)** DLS distribution of exosomes. **(C)** Western blot analysis of surface markers namely, CD9, CD63, and CD81 of exosomes derived from the MSCs and the control MSCs lysates (*n* = 3). **(D)** The surface markers CD105, CD90, and CD73 of MSCs were verified in comparison to the IgG control **p < 0.01, compared with the control. **(E)** Evaluation of circRNA_0002113 expression after 1 h of anoxia/8 h of reoxygenation followed by treatment with exosomes isolated from pLVXcirc treated MSCs. **(F,G)** The protein expression of RUNX1 after 1 h of anoxia/8 h of reoxygenation followed by treatment with exosomes isolated from bone marrow-derived MSCs. **(H,I)** TUNEL staining and quantification of TUNEL positive cells after 1 h of anoxia/8 h of reoxygenation followed by treatment with exosomes isolated from pLVXcirc treated MSCs, Scale bar: 20 μm. **(J)** The co-labeled RUNX1 and DAPI were estimated in H9C2 cells after 1 h of anoxia/8 h of reoxygenation followed by treatment with exosomes isolated from pLVXcirc treated MSCs, Scale bar: 25 μm. **(K)** The number of RUNX1 positive cells was determined following different treatments. *p < 0.05, **p < 0.01, compared with the control. #*p* < 0.05, ##*p* < 0.01, compared with the A/R group.

To explore the effect of exosomes derived from MSCs on the A/R H9C2 cell apoptosis, exosomes isolated from pLVXcirc treated MSCs were used to treat A/R H9C2 cells. Morphology and size of exosomes derived from the pLVXcirc treated MSCs were determined using TEM (Figure 2A). The diameter of exosomes was testified by DLS (Figure 2B). The surface markers namely, CD9, CD63, and CD81 of exosomes were verified in comparison to the control MSCs lysates (Figure 2C). The surface markers of MSCs (CD105, CD90, and CD73) were verified in comparison to the control (Figure 2D). The MSCs derived exosomes remained stable for 4 days. The size of the exosomes ranged from 24.5 to 32.9 nm, and the approximate charge was −12.6 mV to −18.3 mV (Table 1). The circRNA_0002113 and RUNX1 expression levels significantly increased, with or without exosomes isolated from MSCs, in A/R H9C2 cells when compared with the control (Figures 2E–G). The normal H9C2 cells were used as a control. As expected, after pLVXcirc treatment, the expression level of circRNA_0002113 and RUNX1 significantly decreased in A/R H9C2 cells when compared with the control (Figures 2E–G). On a similar note, the exosomes derived from pLVXcirc treated MSCs also had decreased circRNA_0002113 and RUNX1 expression (Figures 2E–G). TUNEL staining was used to detect H9C2 cell apoptosis in the A/R model following either pLVXcirc treatment or treatment with exosomes isolated from pLVXcirc infected MSCs. The results indicate that treatment with both, pLVXcirc or exosomes isolated from pLVXcirc infected MSCs significantly decreased cell apoptosis when compared with the A/R control group (Figures 2H,I). This suggests that circRNA_0002113 might induce apoptosis in A/R H9C2 cells.To explore the effect of exosomes isolated from pLVXcirc treated MSCs on the location and expression of RUNX1, immunofluorescence staining of RUNX1 was carried out in A/R H9C2 cells. The results reveal that RUNX1 was mainly located in the nucleus of H9C2 cells (Figure 2J). Moreover, RUNX1 immunoreactivity was enhanced in A/R H9C2 cells, with or without exosomes isolated from MSCs (Figures 2J,K). Most importantly, after treatment with pLVXcirc or exosomes isolated from pLVXcirc treated MSCs, RUNX1 nuclear translocation was found to be inhibited in A/R H9C2 cells (Figure 2J). These results suggest that exosomes derived from pLVXcirc treated MSCs suppressed RUNX1 nuclear translocation in A/R H9C2 cells.

**TABLE 1:** Identification of exosomes derived from MSCs.

Particles/days	DLS hydrodynamic (nm)				
	0	1	2	3	4
Exosome	29.8 (±2.8)	32.9 (±6.5)	31.2 (±3.2)	31.8 (±4.1)	24.5 (±4.4)

Particles/days	Zeta potential (mV)				
	0	1	2	3	4
Exosome	−14.3 (±1.2)	−18.3 (±3.4)	−14.3 (±2.9)	−12.6 (±3.9)	−16.3 (±3.1)

DLS, dynamic light scattering.

### Exosomes Derived From miR-188-3p Treated MSCs Reduced Apoptosis in A/R H9C2 Cells

**FIGURE 3:**
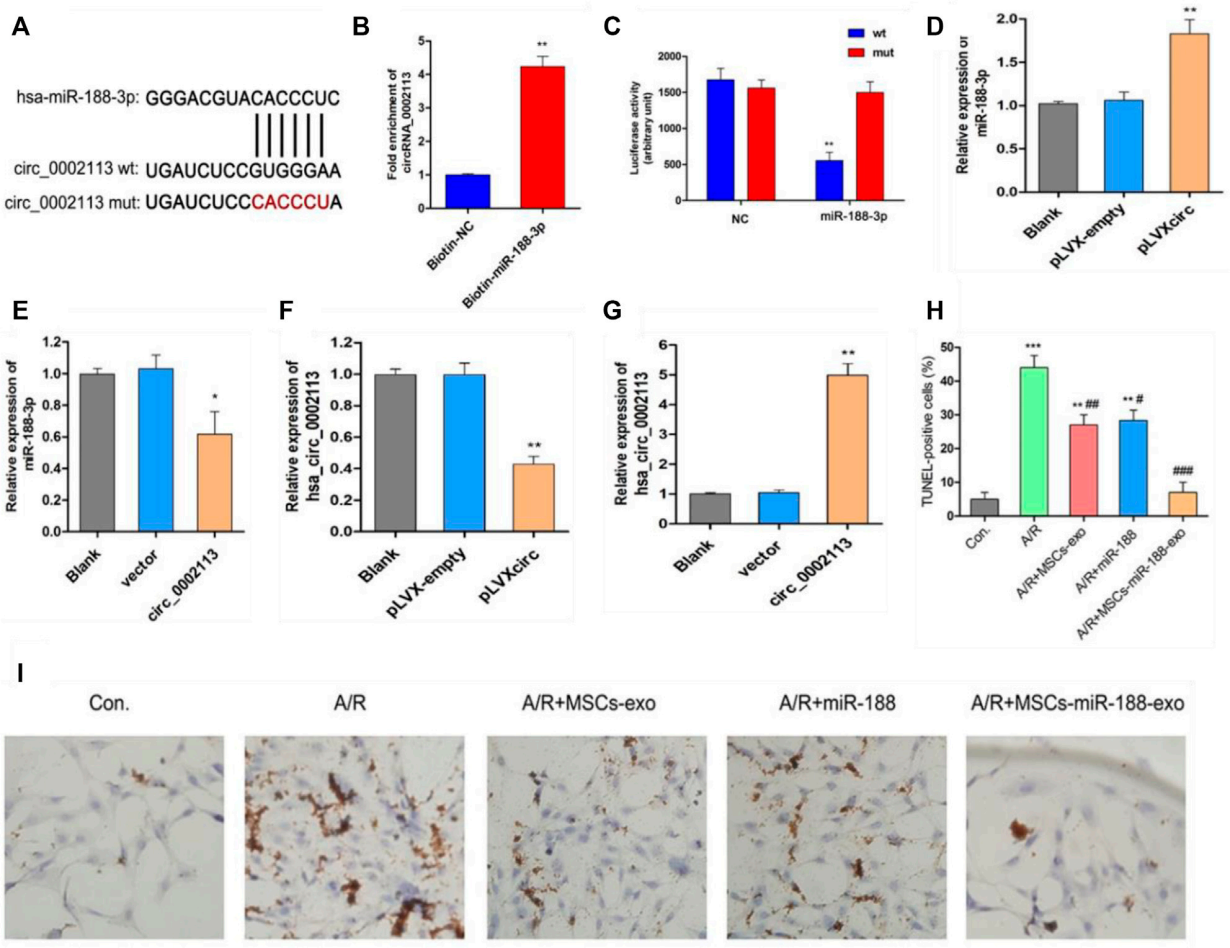
Exosomes derived from miR-188-3p treated MSCs decreased apoptosis in A/R H9C2 cells. **(A)** The potential interaction sequence between circRNA_0002113 and miR-188-3p as identified by the CircInteractome program. **(B)** The RNA pull-down assay and **(C)** dual-luciferase reporter assay were used to determine the interaction of circRNA_0002113 with miR-188-3p in H9C2 cells. The expression levels of miR-188-3p were determined following **(D)** down-regulation or **(E)** up-regulation of circRNA_0002113. The expression levels of circRNA_0002113 were testified following **(F)** down-regulation or **(G)** up-regulation of circRNA_0002113. **(H,I)** TUNEL staining and quantification of TUNEL positive cells after 1 h of anoxia and 8 h of reoxygenation following treatment with miR-188-3p or exosomes isolated from miR-188-3p treated MSCs, Scale bar: 20 μm *p < 0.05, **p < 0.01, ***p < 0.001, compared with the control. #*p* < 0.05, ##*p* < 0.01, ###*p* < 0.001, compared with the A/R group.

miR-188-3p was identified as the miRNA target of circRNA_0002113 by the CircInteractome program (Figure 3A). This interaction will help to explore the mechanism of circRNA_0002113 in regulating the H9C2 cell apoptosis. Previous studies indicate that miR-188-3p is down-regulated in myocardial infarction(Wang et al., 2015). To validate the interaction between circRNA_0002113 and miR-188-3p, a biotin-labeled miR-188-3p probe pull-down assay was performed in H9C2 cells. The expression of circRNA_0002113 was significantly high in the miR-188-3p probe treated precipitates when compared with the control (Figure 3B). To further verify the relationship between circRNA_0002113 and miR-188-3p in cardiomyocytes, the dual-luciferase reporter assay was performed following treatment with miR-188-3p mimics, using *wt* and *mut* type of circRNA_0002113 luciferase reporters. The results reveal that treatment with miR-188-3p mimics drastically reduced the luciferase activity of *wt* of circRNA_0002113 region, but not *mut* type of circRNA_0002113 region (Figure 3C). This is indicative of the potential interaction between miR-188-3p and circRNA_0002113 in H9C2 cells. Moreover, miR-188-3p expression was significantly increased after the pLVXcirc treatment (Figure 3D), while significantly decreased following treatment with circRNA_0002113 over-expression vector (Figure 3E). The expression levels of circRNA_0002113 in knocked-down and overexpressing H9C2 cells were shown in Figures 3F,G. TUNEL staining was used to investigate the effect of, treatment with miR-188-3p or exosomes isolated from miR-188-3p treated MSCs, on H9C2 cell apoptosis in the A/R cell model. The results reveal that cell apoptosis was significantly enhanced in A/R H9C2 cells when compared with the control. In contrast, treatment with miR-188-3p or exosomes isolated from miR-188-3p treated MSCs, significantly decreased H9C2 cell apoptosis when compared with the A/R group (Figures 3H,I). Meanwhile, exosomes isolated from MSCs treatment also significantly reduced the H9C2 cell apoptosis when compared with the A/R group (Figures 3H,I).

### CircRNA_0002113 Modulates RUNX1 Nuclear Translocation by Sponging miR-188-3p in A/R H9C2 Cells

**FIGURE 4:**
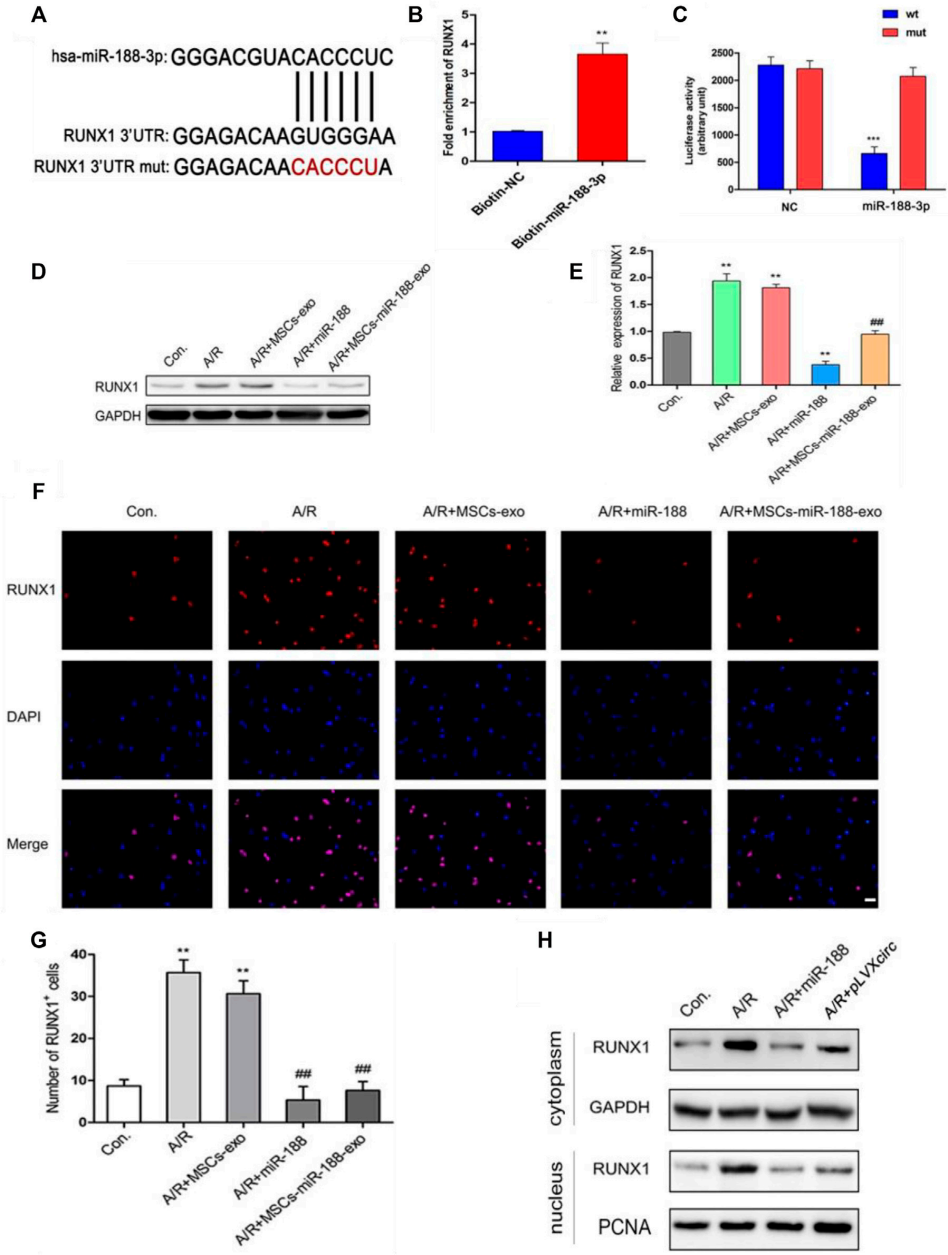
miR-188-3p suppressed RUNX1 nuclear translocation in A/R H9C2 cells. **(A)** The potential interaction sequence between miR-188-3p and RUNX1 as identified by the target prediction program. **(B)** The RNA pull-down assay and **(C)** dual-luciferase reporter assay were used to determine the interaction of miR-188-3p with RUNX1 in H9C2 cells. **(D,E)** The RUNX1 protein expression after 1 h of anoxia and 8 h reoxygenation following different treatments. **(F)** Co-labeled RUNX1 and DAPI in H9C2 cells after 1 h of anoxia and 8 h of reoxygenation following different treatments. Scale bar: 25 μm. **(G)** The number of RUNX1 positive cells was determined following different treatments. **(H)** RUNX1 protein levels in cytoplasm and nucleus following either miR-188-3p up-regulation or circRNA_0002113 down-regulation. **p < 0.01, ***p < 0.001, compared with the control. #*p* < 0.05, ##*p* < 0.01, ###*p* < 0.001, compared with the A/R group.

To further explore the mechanism of miR-188-3p regulating the apoptosis of A/R cells, H9C2 cells were treated with miR-188-3p or exosomes isolated from miR-188-3p transfected MSCs. Using the miRNA target prediction program analysis, miR-188-3p was predicted to target the 3′UTR of RUNX1 (Figure 4A). To validate the relationship between miR-188-3p and RUNX1, a biotin-labeled miR-188-3p probe pull-down assay was performed in H9C2 cells. The RUNX1 expression was significantly high in the miR-188-3p probe treated precipitates when compared with the control (Figure 4B). To further verify the relationship between RUNX1 and miR-188-3p in cardiomyocytes, the dual-luciferase reporter assay was performed following treatment with miR-188-3p mimics or negative control, using *wt* and *mut* type of 3′UTR of RUNX1 luciferase reporters. The results reveal that treatment with miR-188-3p mimics significantly decreased the luciferase activity of *wt* of 3′UTR of RUNX1 but not of the *mut* type when compared with the control (Figure 4C). This implicates that miR-188-3p directly targets RUNX1 in H9C2 cells. RUNX1 expression was dramatically enhanced in the A/R cell model. However, following treatment with miR-188-3p or exosomes isolated from miR-188-3p treated MSCs, the RUNX1 expressions reduced in A/R H9C2 cells when compared with the normal H9C2 cells (Figures 4D,E). To explore the role of miR-188-3p in modulating RUNX1 nuclear translocation in A/R H9C2 cells, the co-labeled RUNX1 and DAPI were estimated following treatment with exosomes isolated from miR-188-3p treated MSCs. The results exhibit that RUNX1 and DAPI positive cells significantly increased in A/R H9C2 cells with or without exosomes isolated from MSCs treatment (Figures 4F,G). Interestingly, treatment of A/R H9C2 cells with either miR-188-3p or exosomes isolated from miR-188-3p treated MSCs suppressed RUNX1 nuclear translocation (Figure 4F). Moreover, both up-regulation of miR-188-3p and down-regulation of circRNA_0002113 suppressed the translocation of RUNX1 protein from the cytoplasm to the nucleus (Figure 4H).

### CircRNA_0002113/miR-188-3p/RUNX1 Axis Mediates A/R H9C2 Cell Apoptosis by Regulating the USP7/p53 Pathway

**FIGURE 5:**
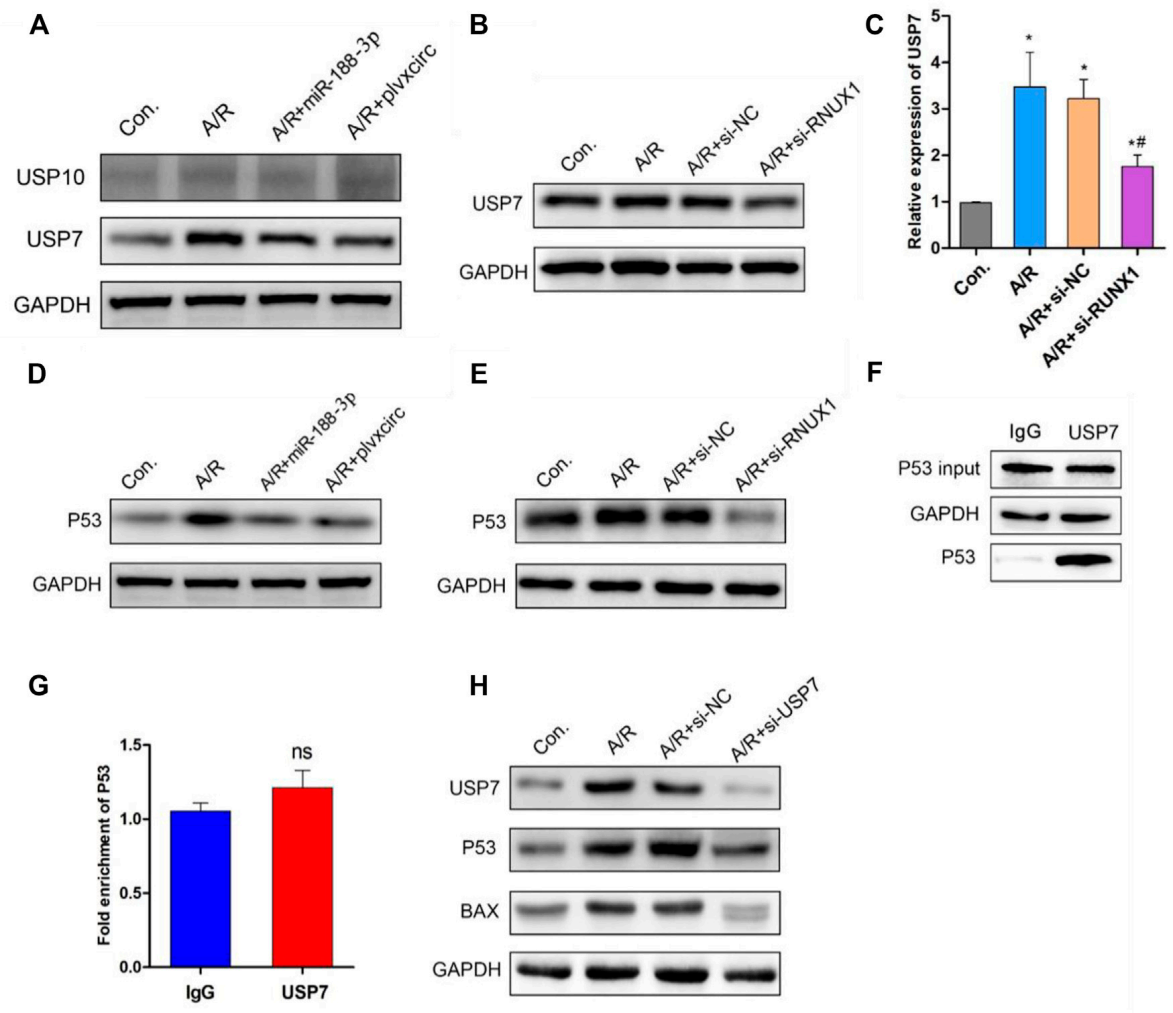
Down-regulation of RUNX1 inhibits cell apoptosis by regulating the USP7/p53 pathway in A/R H9C2 cells. **(A)** The USP7 and USP10 protein expression were determined following miR-188-3p up-regulation or circRNA_0002113 down-regulation. **(B)** The mRNA and **(C)** protein expression of USP7 was evaluated following down-regulation of RUNX1. **(D)** The p53 protein expression evaluated after miR-188-3p up-regulation or circRNA_0002113 down-regulation. **(E)** The p53 protein expression was evaluated following down-regulation of RUNX1. **(F)** Co-immunoprecipitation assay was employed to investigate the relationship between USP7 and p53. **(G)** ChIP analysis to demonstrate the binding capacity of USP7 with p53 in H9C2 cells. **(H)** p53 and BAX protein expressions evaluated following down-regulation of USP7. *p < 0.05, compared with the control. ^#^ *p* < 0.05, compared with the A/R group.

Previous studies have demonstrated that the deubiquitinating enzyme signaling pathway was involved in apoptotic protein degradation in myocardial damage (Singh et al., 2016; Rossi et al., 2017). Following up-regulation of miR-188-3p or down-regulation of circRNA_0002113 in H9C2 cells, the deubiquitinating enzyme signaling pathway involved in apoptotic protein degradation was investigated. The results indicate that ubiquitin-specific protease 7 (USP7) protein expression was decreased, whereas ubiquitin-specific protease 10 (USP10) protein expression was unaffected (Figure 5A). This suggests the involvement of USP7 in circRNA_0002113/miR-188-3p/RUNX1 axis mediated alleviation of A/R H9C2 cell apoptosis. To explore the effect of RUNX1 on USP7 expression, RUNX1 was knock-down by transfection of si-RUNX1 in A/R H9C2 cells. As expected, the mRNA and protein expression levels of USP7 decreased dramatically following the down-regulation of RUNX1 (Figures 5B,C). To further investigate the relationship between USP7 and apoptotic proteins, the potential interaction between USP7 and p53 was evaluated in H9C2 cells. First, we found that p53 protein expression dramatically decreased following up-regulation of miR-188-3p or down-regulation of circRNA_0002113 and RUNX1 (Figures 5D,E). The results of Co-IP analysis demonstrated that the binding capacity of USP7 with p53 was increased in H9C2 cells (Figure 5F). The Co-IP analysis also showed the amount of immunoprecipitated protein of p53 was increased upon treatment of USP7 antibody (Figure 5F). The results indicate that USP7 elevates the protein expression of p53 by directly binding to p53 protein. However, the ChIP assay showed that USP7 hardly interacted with p53 in chromatin levels (Figure 5G), suggesting that USP7 regulated p53 expression in the post-translational stage. Moreover, p53 and BAX protein expression level was reduced following USP7 knockdown (Figure 5H). These findings suggest that USP7 regulates the A/R H9C2 cell apoptosis through the p53 pathway.

### Exosomes Derived From pLVXcirc Treated MSCs Inhibited Myocardial Infarction in I/R Rats *in vivo*

**FIGURE 6:**
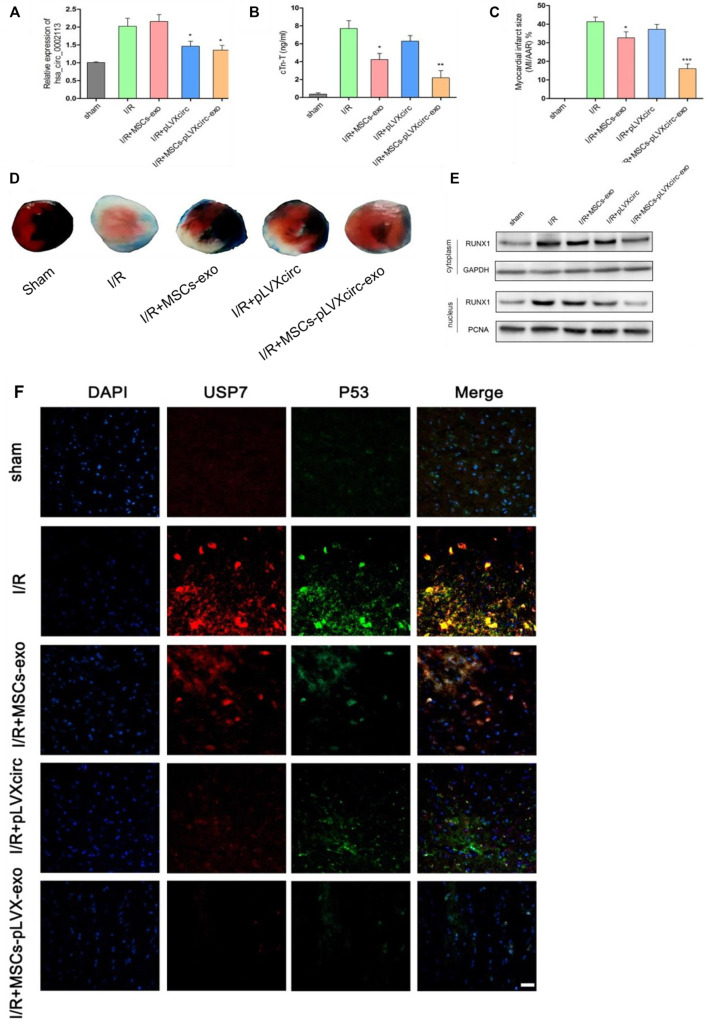
Exosomes derived from pLVXcirc treated MSCs reduced the infarction size 4 weeks post-surgery. **(A)** The circRNA_0002113 expression was evaluated after treatment with exosomes derived from pLVXcirc treated MSCs (*n* = 5). **(B)** Exosomes derived from pLVXcirc treated MSCs decreased cTn-T content after 4 weeks post-surgery (*n* = 5). **(C,D)** The TTC/Evan blue staining and quantitative infarction size 4 weeks post-surgery after treatment with exosomes isolated from pLVXcirc treated MSCs (*n* = 5). **(E)** The cytoplasmic and nuclear RUNX1 protein expression levels were evaluated in I/R hearts following treatment with exosomes isolated from pLVXcirc treated MSCs (*n* = 5). **(F)** The co-labeled USP7 and p53 were estimated in I/R hearts following treatment with exosomes isolated from pLVXcirc treated MSCs. Scale bar: 50 μm *p < 0.05, **p < 0.01, ***p < 0.001, compared with I/R group.

I/R rat models were treated with pLVXcirc or exosomes derived from pLVXcirc infected MSCs, to explore the effect of these treatments on myocardial infarction *in vivo*. The result exhibited that circRNA_0002113 expression level significantly decreased after treatment with pLVXcirc or exosomes isolated from pLVXcirc infected MSCs when compared with the I/R group (Figure 6A). Moreover, cTn-T, the marker of myocardial infarction, decreased significantly after treatment with exosomes isolated from pLVXcirc infected MSCs, reduced slightly after treatment with exosomes isolated from MSCs, whereas the cTn-T content did not significantly change after treatment with pLVXcirc when compared with the control (Figure 6B). The infarct size, as determined by the TTC/Evan blue staining, significantly decreased after treatment with exosomes isolated from pLVXcirc infected MSCs whereas the infarct size was only slightly reduced after treatment with exosomes isolated from MSCs when compared with the I/R rats (Figures 6C,D). The RUNX1 protein expression increased in I/R rats, but not in the group treated with exosomes isolated from pLVXcirc treated MSCs (Figure 6E). Consistent with the *in vitro* results, up-regulation of miR-188-3p or down-regulation of circRNA_0002113 inhibited the expression of RUNX1 protein in the nucleus of I/R rat hearts tissues (Figure 6E). To explore the interaction between USP7 and p53 in I/R rats, co-labeled USP7 and p53 staining were estimated following treatment with exosomes isolated from pLVXcirc infected MSCs. The fluorescence intensity of USP7 and p53 was barely detected in sham group. In I/R group, the fluorescence intensity of USP7 and p53 was dramatically enhanced, and the co-labeled USP7 and p53 staining were testified in the I/R rats, indicating that USP7 might regulate p53 in I/R hearts (Figure 6F). In addition, the fluorescence intensity of USP7 and p53 reduced following treatment with exosomes isolated from pLVXcirc infected MSCs (Figure 6F). These results suggest that exosomes derived from pLVXcirc treated MSCs, suppress the USP7/p53 interaction and thereby inhibit myocardial infarction in I/R rats *in vivo*.

## Discussion

MSCs transplantation reduces the area of myocardial congestion and thereby improves left ventricular ejection fraction, increases blood vessel density, and maintains cardiac function. Hence, MSCs transplantation has become a hotspot in cardiac regeneration (Dakhlallah et al., 2015; Singh et al., 2016; Rossi et al., 2017). Previous studies in the rat model have shown that MSCs transplantation improves cardiac function after acute myocardial infarction (Mao et al., 2017; Omar et al., 2019). However, transplanted MSCs did not efficiently transform into cardiomyocytes and myocardial tissue, and <6% of transplanted MSCs existed for 2 weeks post-transplantation (Freyman et al., 2006). This suggests the involvement of novel pathways in the myocardial repair process. Accumulating evidence reveals that exosomes derived from MSCs exert the biological effects of reducing heart damage (Safari et al., 2016; Wang et al., 2018). Ibrahim et al. showed that exosomes secreted by human-derived cardiomyocytes reduced cardiomyocyte apoptosis, and promoted cardiomyocyte proliferation and angiogenesis by the role of exosomal miR-146 (Ibrahim et al., 2014). Khan et al. showed that embryonic stem cell-derived exosomes promoted angiogenesis, enhanced survival of cardiomyocytes, and promoted survival and proliferation of cardiac progenitor cells by exosomal miR-294 in the acute myocardial infarction model (Khan et al., 2015). In this study, we found that exosomes derived from MSCs reduced cell apoptosis in the A/R cell model *in vitro*. However, the detailed molecular mechanism of these exosomes derived from MSCs regulating A/R cell apoptosis is yet to be elucidated.

A previous study demonstrates that exosomes secreted by GATA-4 over-expressing MSCs, increase cardiomyocyte survival, reduce cardiomyocyte apoptosis, and preserves mitochondrial membrane potential in cardiomyocytes cultured under a hypoxic environment (Yu et al., 2015). A recent study revealed that exosomal miR-21, derived from human endometrium MSCs, effectively enhanced cardioprotection both, *in vitro* and *in vivo* (Wang et al., 2017). Teng Ma *et al.* found that transplantation of exosomal miR-132 derived from MSCs markedly enhanced neovascularization in the peri-infarct zone and preserved the heart of ischemic mice (Ma et al., 2018). In addition, Changchen Xiao *et al.* demonstrated that MSCs transplantation improved autophagic flux after myocardial infarction by the function of exosomal miR-125b (Xiao et al., 2018). This study aimed to explore the effect and molecular mechanism of exosomes derived from MSCs in regulating A/R cell function. To achieve this aim, circRNA_0002113 was down-regulated and its tentative interaction target, miR-188-3p was up-regulated in A/R cells. Interestingly, exosomes derived from both, MSCs without circRNA_0002113 and miR-188-3p treated MSCs, reduced cell apoptosis proportion in A/R cells. On the other hand, down-regulation of circRNA_0002113 or up-regulation of miR-188-3p suppressed RUNX1 nuclear translocation in A/R cells. This suggests that exosomes derived from both, MSCs without circRNA_0002113 and miR-188-3p treated MSCs, reduce myocardial injury by inhibiting nuclear translocation of RUNX1 *in vitro*. In addition, exosomes derived from MSCs without circRNA_0002113 brought about a significant reduction in the infarct size in I/R rat models when compared with exosomes derived from MSCs. This implicates that exosomes from MSCs might have other signaling pathways in the treatment of myocardial infarction *in vivo*.

The deubiquitinating enzyme signaling pathway is reported to play an important role in the development and progression of myocardial infarction (Cilenti et al., 2011). A previous study has shown that USP7 exacerbated myocardial ischemic injury by promoting inflammation and apoptosis of cardiomyocytes (Xue et al., 2021). In this study, we found that USP7 interacts with p53 protein. This suggests that USP7 regulated the circRNA_0002113/miR-188-3p/RUNX1 axis mediated apoptosis of A/R H9C2 cells through the p53 pathway. Consistent with the *in vitro* results, the USP7/p53 interaction was enhanced in I/R hearts and suppressed following treatment with exosomes derived from pLVXcirc treated MSCs. These results indicate that the circRNA_0002113/miR-188-3p/RUNX1 axis mediated A/R H9C2 cell apoptosis by regulating the USP7/p53 pathway both, *in vitro* and *in vivo*.

## Conclusion

In summary, exosomes derived from MSCs without circRNA_0002113 could suppress myocardial infarction *in vitro* as well as *in vivo*. These findings revealed a tentative molecular mechanism of these exosomes derived from MSCs regulating myocardial infarction *in vitro* and *in vivo*. The therapeutic effects of exosomes derived from circRNA_0002113 MSCs might provide a novel potential manner for the treatment of myocardial infarction in the clinic.

## Data Availability

The raw data supporting the conclusion of this article will be made available by the authors, without undue reservation.
